# Measurement Properties of Instruments Assessing Digital Competence in Nursing: A Systematic Review

**DOI:** 10.1055/a-2780-7093

**Published:** 2026-01-22

**Authors:** Fabio D'Agostino, Ilaria Erba, Elske Ammenwerth, Vered Robinzon, Gad Segal, Nissim Harel, Elisabetta Corvo, Refael Barkan, Hadas Lewy, Noemi Giannetta

**Affiliations:** 1 Department of Medicine, UniCamillus, Saint Camillus International University of Health Sciences, Rome, Italy; 2 Bambino Gesù Children's Hospital, IRCCS, Rome, Italy; 3 UMIT TIROL, Private University for Health Sciences and Health Technology, Hall in Tirol, Austria; 4 Sheba Medical Center, Tel-Hashomer, Ramat Gan, Israel; 5 Department of Health Systems Management, School of Health Sciences, Ariel University, Ariel, Israel; 6 The Gray Faculty for Healthcare Science and Medicine, Tel-Aviv University, Tel-Aviv, Israel; 7 Department of Software Engineering, Azrieli College of Engineering, Jerusalem, Israel; 8 Principal Lecturer, School of Nursing, Midwifery, Allied and Public Health - Canterbury Christ Church University, United Kingdom; 9 Advisor, CNAI - Consociazione Nazionale infermieri/e - Italian Nursing Association, Italy; 10 Entrepreneurship and Internationalization HIT-Holon Institute of Technology, Holon, Israel; 11 Department of Digital Medical Technologies, HIT, Holon Institute of Technology, Holon, Israel

**Keywords:** digital competence, instruments, nursing informatics, psychometrics, systematic review

## Abstract

**Background:**

The digital transformation of healthcare is reshaping care delivery among healthcare professionals, requiring nurses to develop digital competencies. These competencies are essential but often underdeveloped due to limited training and resources. Global initiatives emphasize integrating these competencies into nursing education, necessitating valid instruments to assess them.

**Objective:**

This systematic review aims to identify instruments measuring digital competence in nursing and to assess their measurement properties.

**Methods:**

This review was registered in PROSPERO (identifier: CRD42024522349) and conducted according to PRISMA guidelines. A systematic search was performed in CINAHL, PubMed/MEDLINE, and Scopus on instruments assessing digital competencies in nursing and reporting measurement properties. Measurement properties and their methodological quality were assessed using the COSMIN criteria, and the overall quality of the evidence was graded using a modified GRADE approach.

**Results:**

A total of 27 instruments were identified, relating to three interconnected constructs: nursing informatics, digital health, and information and communication technology. Based on their measurement properties, the instruments were categorized into three groups (A, B, C) following the COSMIN methodology to support recommendations for use. Six instruments were classified under category A (recommended for use): the DigiHealthCom and DigiComInf instruments, the Turkish version of TANIC, the short version of ITASH, the Digital Competence Questionnaire, and the 30-item Arabic version of SANICS. Twenty instruments were categorized under category B (potentially recommendable, but further validation is needed). One instrument was placed in category C (not recommended for use).

**Conclusion:**

As digital competence becomes an increasing priority in education and public health, valid and reliable instruments are essential for assessing and monitoring these competencies. Such instruments support the identification of training needs, the evaluation of educational outcomes, and the integration of digital skills into nursing curricula and clinical practice, ultimately strengthening the digital readiness of the nursing workforce.

## Introduction

The digitalization process happening in the healthcare sector is affecting the care that healthcare professionals deliver to patients and how they communicate in the healthcare system to provide continuity of care. 1 This process requires healthcare providers to acquire new knowledge and skills to deliver care using digital health technologies. 2 Healthcare professionals, such as nurses at various levels, must be able to gather, analyze, use, and disseminate data and information about patient care. This ability is essential for nurses to effectively fulfill their healthcare roles. 3

Information and communication technology (ICT) is a broad field encompassing all technologies used to manage and communicate information across various sectors, including healthcare, education, and business. Within this context, digital health and nursing informatics (NI) emerge as specialized subsets of ICT, with digital health focusing on the application of ICTs in healthcare, and NI concentrating on their use within nursing practice and science. Digital health encompasses the technical, methodological, social, and personal competencies required to engage effectively within the digital healthcare environment. 4 NI is an evolving field that can help to streamline and optimize the integration of information technology and processes into healthcare practices. 5 6 NI is a specialty that integrates nursing, information, and computer sciences to improve people's health. 7 8 9

Since its early conceptualization by Graves and Corcoran 10 and further developments by Ball, 11 Hannah et al, 12 Saba, 13 the Technology Informatics Guiding Education Reform (TIGER) Initiative, 14 15 and many others, NI has evolved from data processing into an integrated yet distinct discipline within the broader field of digital health. It builds on the data–information–knowledge–wisdom framework, extending general digital health and ICT competencies to include topics such as nursing reasoning along the nursing process, ethical data use, and patient-centered decision-making. Within this framework, nurses move beyond functional IT literacy toward transforming data into knowledge to support safe and evidence-based nursing care.

NI competencies are the knowledge, skills, and attitudes nurses need to develop, implement, and manage ICTs like electronic health records and telehealth, providing patient-centered digital care and interaction. 16 17 18 19 20 21 While general digital health and ICT competencies are relevant for all healthcare professionals, NI competencies are specific to the nursing role, integrating nursing science with informatics knowledge to support patient-centered care, clinical decision-making, and safe and effective use of digital tools within nursing practice. Recent global policies, including the WHO Global Strategy on Digital Health 2020 to 2025 22 and the EU Digital Skills and Jobs Initiative, 23 emphasize the importance of a digitally competent health workforce, further highlighting the critical role of NI in achieving these goals. 22 23

These competencies are necessary to accomplish job responsibilities in the current healthcare arena. 15 Unfortunately, challenges exist in these competencies, such as a lack of training, limited access to educational resources, and low confidence among nurses in using digital tools effectively. 24 25 For example, le Roux et al 26 found that the majority of professional nurses rated their NI competencies as limited.

Worldwide guidelines and initiatives promote the development of curricula aimed at improving digital competencies, recognizing these as core competencies 27 28 in nursing education. Nursing education highlights the importance of these competencies as fundamental skills, with ongoing efforts to enhance them through targeted educational initiatives. 27 29 To support these initiatives, valid and reliable assessment instruments to measure these competencies are needed.

These instruments can support the evaluation of training effectiveness and help identify areas for improvement to guide future educational strategies. However, despite decades of conceptual and curricular development, the empirical evidence on the validity and reliability of available instruments remains fragmented. While some literature reviews exist 18 30 a comprehensive systematic review is still lacking, highlighting the need for a structured synthesis of the available evidence. Li et al 31 conducted a systematic review of instruments for NI competencies, but it did not address the broader constructs of ICT and digital health. A recent scoping review highlighted how digital health competence is assessed in healthcare professionals, including nurses. 4 In our review, we include NI alongside the related constructs of ICT and digital health, as instruments have been developed to assess competencies across all three areas. Considering all three constructs provides insight into how conceptually similar competencies have been defined and measured under different labels across the literature.

## Objective

The aim of our systematic review was to identify instruments related to NI and its broader concepts, such as ICT and digital health, used to assess digital competences among nursing stakeholders, and to evaluate their measurement properties. Research questions were: (1) What instruments grounded in NI, ICT, or digital health have been used to assess digital competence among nursing stakeholders? (2) Which instruments can be recommended for use based on the quality of the available evidence regarding their measurement properties?

## Methods

This study has been registered in the International Prospective Register of Systematic Reviews (PROSPERO; identifier: CRD42024522349) and was reported according to the Preferred Reporting Items for Systematic Reviews and Meta-analyses (PRISMA) guidelines. 32 A systematic review was conducted using the following databases: CINAHL, PubMed/MEDLINE, and Scopus. We used search terms ( Table 1 ), and we considered the peer review of all the search strategies used in the databases. The research question was structured using the PIO format 33 : P: nurses (e.g., nurses, nursing students, and nurse leaders); I: instruments related to NI and its broader concepts; O: measurement properties (e.g., validity and reliability). The final database search was conducted in March 2025.

**Table 1:** Search strategies used in the three bibliographic databases

**PubMed Medline**
(“Surveys and Questionnaires”[MeSH Terms] OR “scale*”[All Fields] OR “instrument*”[All Fields] OR “tool*”[All Fields] OR “survey”[All Fields]) AND (“Nursing Informatics”[MeSH Terms] OR “nursing informatic*”[All Fields] OR “Information Technology”[MeSH Terms]) AND (“Reproducibility of Results”[MeSH Terms] OR “Dimensional Measurement Accuracy”[MeSH Terms] OR “Sensitivity and Specificity”[MeSH Terms] OR “validity”[All Fields] OR “reliability”[All Fields] OR “psychometric”[All Fields] OR “valid”[All Fields] OR “reliable”[All Fields] OR “sensibility”[All Fields] OR “accuracy”[All Fields] OR “responsiveness”[All Fields] OR “predictive value*”[All Fields] OR “specificity”[All Fields] OR “simplicity”[All Fields] OR “applicability”[All Fields] OR “interpretability”[All Fields] OR “Observer Variation”[MeSH Terms] OR “measurement error*”[All Fields] OR “hypothesis test*”[All Fields] OR “hypotheses test*”[All Fields])
**CINAHL**
((MH “Surveys”) OR (MH “Structured Questionnaires”) OR (AB “Surveys and Questionnaires”) OR (TI “Surveys and Questionnaires”) OR (AB “scale”) OR (TI “scale”) OR (AB “scales”) OR (TI “scales”) OR (AB “instrument”) OR (TI “instrument”) OR (AB “instruments”) OR (TI “instruments”) OR (AB “tool”) OR (TI “tool”) OR (AB “tools”) OR (TI “tools”) OR (AB “survey”) OR (TI “survey”)) AND ((MH “Nursing Informatics”) OR (MH “Informatics Nurses”) OR (AB “Nursing Informatics”) OR (TI”Nursing Informatics”) OR (AB “nursing informatic”) OR (TI “nursing informatic”) OR (AB”Information Technology”) OR (TI”Information Technology”)) AND ((MH “Reproducibility of Results”) OR (AB “Reproducibility of Results”) OR (TI “Reproducibility of Results”) OR (AB “Dimensional Measurement Accuracy”) OR (TI “Dimensional Measurement Accuracy”) OR (MH “Sensitivity and Specificity”) OR (AB “Sensitivity and Specificity”) OR (TI “Sensitivity and Specificity”) OR (MH “Validity”) OR (AB “validity”) OR (TI “validity”) OR (MH “Reliability”) OR (AB “reliability”) OR (TI “reliability”) OR (AB “psychometric”) OR (TI “psychometric”) OR (AB “valid”) OR (TI “valid”) OR (AB “reliable”) OR (TI “reliable”) OR (AB “sensibility”) OR (TI “sensibility”) OR (AB “accuracy”) OR (TI “accuracy”) OR (AB “responsiveness”) OR (TI “responsiveness”) OR (AB “predictive value”) OR (TI “predictive value”) OR (AB “predictive values”) OR (TI “predictive values”) OR (AB “specificity”) OR (TI “specificity”) OR (AB “simplicity”) OR (TI “simplicity”) OR (AB “applicability”) OR (TI “applicability”) OR (AB “interpretability”) OR (TI “interpretability”) OR (AB “Observer Variation”) OR (TI “Observer Variation”) OR (MH “Measurement Error”) OR (AB”measurement error”) OR (TI”measurement error”) OR (AB”measurement errors”) OR (TI”measurement errors”) OR (AB”hypothesis test”) OR (TI “hypothesis test”) OR (AB”hypothesis tests”) OR (TI “hypothesis tests”) OR (AB “hypotheses test”) OR (TI “hypotheses test”) OR (AB “hypotheses tests”) OR (TI “hypotheses tests”))
**Scopus**
(INDEXTERMS (“surveys and questionnaires”) OR TITLE-ABS (“scale”) OR TITLE-ABS (“instrument”)) AND (INDEXTERMS (“nursing informatics”) OR INDEXTERMS (“information technology”)) AND (INDEXTERMS (“reproducibility of results”) OR INDEXTERMS (“dimensional measurement accuracy”) OR INDEXTERMS (“sensitivity and specificity”) OR TITLE-ABS (“validity”) OR TITLE-ABS (“reliability”) OR TITLE-ABS (“psychometric”) OR TITLE-ABS (“sensibility”) OR TITLE-ABS (“accuracy”) OR TITLE-ABS (“responsiveness”) OR TITLE-ABS (“predictive value”) OR TITLE-ABS (“simplicity”) OR TITLE-ABS (“applicability”) OR TITLE-ABS (“interpretability”) OR INDEXTERMS (“observer variation”) OR TITLE-ABS (“measurement error”) OR TITLE-ABS (“hypothesis test”))

Inclusion criteria were: (1) focus on instruments measuring digital competencies in nursing (i.e., digital, NI, or ICT competencies); (2) evaluation of the instrument's measurement properties (e.g., validity, reliability, internal consistency, and responsiveness); (3) original articles or dissertations; (4) no publication date limits; (5) English language.

Exclusion criteria were: (1) instruments not applicable to nurses (e.g., developed only for physicians); (2) protocols or literature reviews; (3) conference abstracts, book chapters, and gray literature. To enhance comprehensiveness, backward citation tracking was performed to identify other versions of included instruments. Additionally, reference lists of relevant reviews were screened, and experts in nursing and health informatics were consulted to identify instruments possibly missed in database searches.

### Screening Procedure

Two researchers with expertise in nursing informatics, instrument measurement properties, and systematic review methodology independently screened the articles to ensure reliability; disagreements were resolved through a third reviewer. Prior to screening, a pilot test on 30 articles was conducted to calibrate the review form, achieving full agreement. Screening was performed using the Rayyan web application. 34

### Data Extraction and Assessment of Methodological Quality

Data extraction was performed independently by two researchers following the Consensus-based Standards for the Selection of Health Measurement Instruments (COSMIN) methodology framework. 35 The COSMIN checklist provides structured criteria to ensure the quality and transparency of measurement evaluations, both for Patient-Reported Outcome Measures and other measurement tools. 36 Using the COSMIN checklist, each measurement property was assessed through predefined standards that allowed judgments on the risk of bias as “very good,” “adequate,” “doubtful,” or “inadequate.” Then, the results of the measurement analyses were rated according to COSMIN guidelines as: sufficient (+): the property was supported by convincing results; insufficient (−): the property was not supported; indeterminate (?): insufficient or unclear information was reported; inconsistent (± ): conflicting results across studies. Finally, the overall quality of the evidence for each measurement property was assessed using the modified GRADE approach, which considers the methodological quality, consistency of results, precision, and directness of evidence. The strength of the evidence was graded as “high,” “moderate,” “low,” or “very low.” 36 37

Two independent evaluators used the COSMIN checklist to assess the measurement properties of the identified instruments and to formulate recommendations regarding their use. 35 All studies were independently assessed by two researchers, and any discrepancies were discussed until full consensus (100%) was achieved. Within this framework, the following information was extracted: authors, year of publication, instrument name, theoretical framework of the instrument, target population, sample size, instrument characteristics, and measurement properties.

## Results

**Fig. 1:**
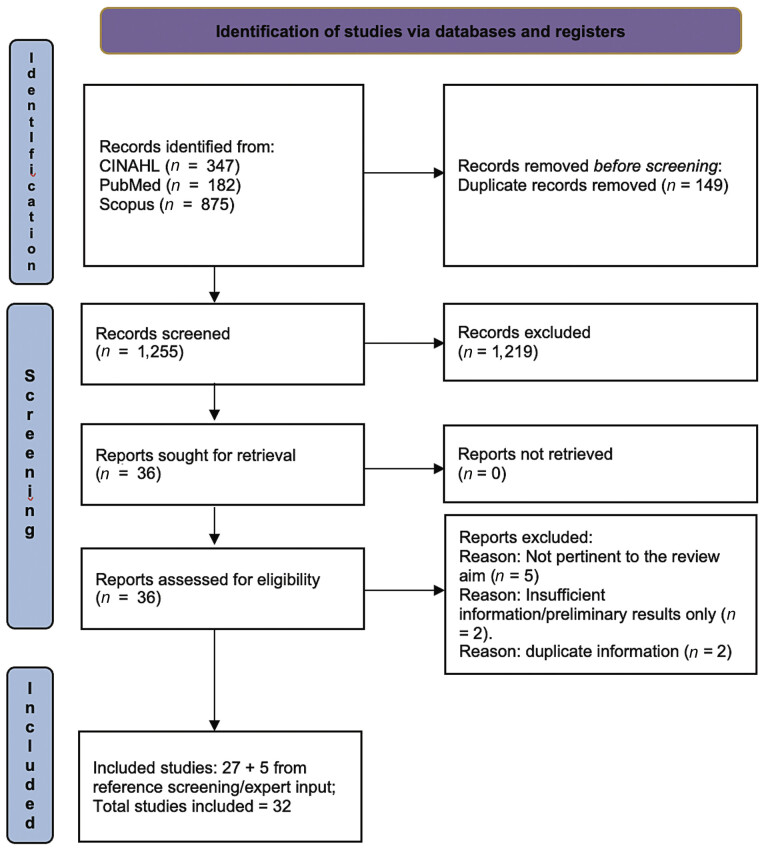
PRISMA 2020 study flow diagram.

The literature search produced a total of 1,404 records (875 in Scopus, 347 in CINAHL, and 182 in PubMed). After removing duplicates, 1,255 records were screened by title and abstract, of which 36 were deemed eligible for full-text review. Following full-text assessment, 27 articles were included in the review, and an additional 5 articles were identified through reference screening and/or expert input. In total, 32 articles were included in the review ( Fig. 1 ).

Twenty-seven instruments were identified. Only four instruments were reported in more than one article, although their different measurement properties were assessed in only one sample. Only the Self-Assessment of Nursing Informatics Competencies Scale (SANICS 30-item) had the same measurement properties assessed in more than one sample. 38 39 40 Most of them ( *n* = 12) were developed in English. The instruments identified were related to three different but related constructs: NI, digital health, and ICT. In the following section, the instruments are presented according to these three constructs. Finally, all the instruments were self-assessment instruments, except for the Digital Competence Test, 41 which was performance-based.

### Nursing Informatics Instruments

Eighteen instruments were identified as measuring NI competencies. Most instruments assessed these competencies in nurses ( *n* = 11), followed by nursing students ( *n* = 5), nurse leaders ( *n* = 1), and informatics nurse specialists ( *n* = 1). Most of the instruments were tested in samples from the United States ( *n* = 7), Canada ( *n* = 3), and South Korea ( *n* = 3). A detailed summary of the instruments' characteristics and their measurement properties is presented in Tables 2 and 3 , respectively (see also Supplementary File , available in the online version only, for measurement values).

**Table 2:** Characteristics of the instruments

Instrument	Framework	Target population	Instruments administration			Mode of administration (self-assessment, performance-based test)	Dimensions, number of items, and response options
			Country	Language	Setting		
Nursing informatics (NI) competence instruments for nurses							
Canadian Nurse Informatics Competency Assessment Scale (C-NICAS), English version 51	Canadian Association of Schools of Nursing (CASN) core entry-to-practice informatics competencies for RNs in Canada (2012)	*n* = 2,844 nurses	Canada	English	NA	Self-assessment	4 Dimensions ● Foundational ICT skills ● Information and knowledge management ● Professional and regulatory accountability ● ICT in the delivery of patient care. 21-item, 4-point Likert scale
Canadian Nurse Informatics Competency Assessment Scale (C-NICAS), French version 46	Canadian Association of Schools of Nursing (CASN) core entry-to-practice informatics competencies for RNs in Canada (2012)	*n* = 25 nurses	Canada	French	Hospital	Self-assessment	4 Dimensions ● Foundational ICT skills ● Information and knowledge management ● Professional and regulatory responsibility ● ICT in the delivery of patient care 21-item, 4-point Likert scale
Korean Nursing Informatics Competence Assessment Scale (K-NICAS) 44	Based on (TIGER) and the nursing informatics competence measurement instrument developed by the Canadian Association of Schools of Nursing	*n* = 214 nurses	South Korea	Korean	Hospital	Self-assessment	5 Dimensions ● Basic ICT use ● Nursing information utilization and management ● Professional responsibility and Ethics ● Use of ICT in nursing ● Attitude toward nursing informatics 20-item, 4-point Likert scale
Nursing Informatics Competencies Questionnaire (NICQ) 42	Master list of competencies developed by Staggers	*n* = 208 nurses	South Korea	Korean	Hospital	Self-assessment	3 Dimensions ● Computer skills ● Informatics knowledge ● Informatics skills. 112-item, 5-point Likert scale
Nursing Informatics Competency Assessment Tool (NICAT) 45	ANA standards (2008), TIGER recommendations (2009), and Benner's Dreyfus model of skill acquisition (1984)	*n* = 27 nurses	USA	English	Hospital	Self-assessment	3 Dimensions ● Computer Literacy ● Informatics literacy ● Information management skills 30-item, 5-point Likert scale
Self-Assessment of Nursing Informatics Competency Scale-18 (T-SANICS 18-item) 49	Based on SANICS, 93-item	*n* = 190 nurses	Turkey	Turkish	Hospital	Self-assessment	3 Dimensions ● Basic computer skills ● Role ● Applied computer skills. 18-item, 5-point Likert scale
The Arabic Self-assessment Nursing Informatics Competency Scale (A-SANICS 30-item) 48	Based on SANICS, 93-item	*n* = 176 nurses	Jordan	Arabic	Hospital	Self-assessment	5 Dimensions ● Clinical informatics roles ● Basic computer knowledge and skills ● Applied computer skills ● Clinical informatics attitude ● Wireless device skills. 30-item, 5-point Likert scale
TIGER-Based Assessment of Nursing Informatics Competencies (TANIC) 16 52	TIGER competencies	*n* = 368 nurses	USA	English	NA	Self-assessment	3 Dimensions ● Information literacy ● Clinical information management ● Basic computer skills. 85-item, 4-point Likert scale
Turkish Version of the TIGER-Based Assessment of Nursing Informatics Competencies (T-TANIC) 47	TIGER competencies	*n* = 518 nurses	Turkey	Turkish	Two university hospitals in Istanbul	Self-assessment	3 Dimensions ● basic computer skills ● clinical information management ● informatics literacy. 85-item, 4-point Likert scale
Unnamed 50	NA	*n* = 205 nurses	Iran	Persian	Hospital	Self-assessment	6 Dimensions ● Self-efficacy ● Evidence-based practice ● Job satisfaction ● Informatics skills ● Computer skills ● Informatics knowledge. 41-item (no response option available)
Unnamed 43	Master list of competencies developed by Staggers	*n* = 197 nurses	Iran	Persian	Hospital	Self-assessment	3 Dimensions ● Basic computer skills ● Informatics ● Informatics Skills 48-item, 4-point Likert scale
Nursing informatics (NI) competence instruments for nursing students							
Canadian Nurse Informatics Competency Assessment Scale-Version 2 (C-NICAS-V2) 55	Based on the Canadian Association of Schools of Nursing (CASN) core entry-to-practice informatics competencies for RNs in Canada (2012) and on C-NICAT v1	*n* = 221 nursing students	Canada	English	University	Self-assessment	4 Dimensions ● Foundational ICT skills ● Information and knowledge management ● Professional responsibility and regulatory accountability ● Use of ICT in the delivery of patient/client care 26-item, 4-point Likert scale
Knowledge, Skills, and Attitudes Toward Nursing Informatics (KSANI) Scale 53	Quality and Safety Education for Nurses (QSEN) Institute informatics competencies for prelicensure Students	*n* = 300 nursing students	USA	English	University	Self-assessment	4 Dimensions ● Educational opportunity to apply informatics ● Knowledge of informatics ● Informatics skills confidence ● Attitude toward informatics. 24-item, 4-point Likert scale
Korean Self-Assessment of Nursing Informatics Competencies Scale (K-SANICS 30-item) 54	Based on SANICS, 93-item	*n* = 254 nursing students	Korea	Korean	University	Self-assessment	6 Dimensions ● Advanced skills for clinical informatics ● Basic application skills ● Basic computer skills ● Roles in nursing informatics ● Skills for using clinical applications ● Attitude about using computers in nursing 30-item, 5-point Likert scale
SANICS 30-item 38 39 40	Based on SANICS, 93-item	*n* = 880 nursing students	USA	English	University	Self-assessment	5 Dimensions ● Basic computer knowledge and skills ● Applied computer skills: clinical informatics ● Clinical informatics role ● Clinical informatics attitudes ● Data/information management skills 30-item, 5-point Likert scale
Self-Assessment of Nursing Informatics Competencies Scale (SANICS 18-item) 56	Based on SANICS, 93-item	*n* = 603 nursing students	USA	English	University	Self-assessment	Unidimensional 18-item, 4-point Likert scale
NI instruments for nurse leaders							
Nursing Informatics Competency Assessment for the Nurse Leader (NICA-NL) 57 58	NA	*n* = 398 nurse leaders	USA	English	NA	Self-assessment	6 Dimensions ● Strategic implementation management ● Advanced information management and education ● Executive planning ● Ethical and legal concepts ● Information systems concepts ● Requirements and system selection 26-item, 6-point Likert scale
NI Competence Instruments for Informatics Nurse Specialists							
The Nursing Informatics Competency Assessment L3/L4 (NICA: L3/L4) 59	TIGER competencies	*n* = 88 informatics nurse specialists	USA	English	NA	Self-assessment	3 Dimensions ● Computer skills ● Informatics knowledge ● Informatics skills. 178-item, 5-point Likert scale
Digital health competence instruments							
DigiComInf 60	NA	*n* = 817 healthcare professionals	Finland	Finnish	Healthcare District and Hospital	Self-assessment	3 Dimensions ● Support from management, ● Organisational practices as part of digital competence development ● Colleagues' adoption and influence 15-item, 4-point Likert scale
DigiHealthCom 60	NA	*n* = 817 healthcare professionals	Finland	Finnish	Healthcare District and Hospital	Self-assessment	5 Dimensions ● Human-centred remote counselling competence ● Digital solutions as part of work ● ICT competence ● Competence in utilising and evaluating digital solutions ● Ethical competence related to digital solutions. 42-item, 4-point Likert scale
Digital Competence Questionnaire (DCQ) 61 63	Framework of digital competence by Golz et al (2023)	*n* = 185 nurses	International	English	NA	Self-assessment	2 Dimensions ● Knowledge, skills ● Attitude toward digital competence. 12-item, 5-point Likert scale
Knowledge, attitudes, and practices (KAP) on digital health 62	NA	*n* = 20 Nurses	Sri Lanka	Sinhala	Hospital	Self-assessment	3 Dimensions ● Knowledge ● Attitude ● Practice 38-item, knowledge: yes, no, uncertain; attitude: 5-point Likert scale; Practice: nonuniform Likert
The digital competence test 41	The ACTIC 2-intermediate level certificate	*n* = 803 healthcare professionals	Spain	Spanish	Healthcare District	Performance-based test	2 “real-life” scenarios with 7 and 11 questions, 4 possible answers
Unnamed 64	Informatics Competencies for Public Health Professionals and the Informatics Competency Domain of Local Health Departments	*n* = 174 healthcare professionals	USA	English	Healthcare District	Self-assessment	2 Dimensions ● Effective use of information ● Effective use of IT. 10-item, 5-point Likert scale
Attitudes toward information and communication technology instruments							
Shortened Version-Information Technology Attitude Scales for Health (ITASH-sv) 65	NA	*n* = 162 nursing students	South Korea	Korean	University	Self-assessment	4 Dimensions ● Care value of ICT ● Training of ICT skills ● ICT confidence ● Workload value of ICT. 21-item, 4-point Likert scale
Technology Attitude Survey 66	NA	*n* = 743 nursing students	USA	English	University	Self-assessment	2 Dimensions ● Confidence in and the benefits of using technology ● Lack of self-efficacy in the use of technology 15-item, 6-point Likert scale
Turkish Version of the Technology Attitude Survey 67	NA	*n* = 238 nursing students	Turkey	Turkish	University	Self-assessment	2 Dimensions ● Confidence in and the benefits of using technology ● Lack of self-efficacy in the use of Technology 15-item, 6-point Likert scale

Abbreviations: ICT, information communication technology; *n* , number; NA, not assessable; TIGER, Technology Informatics Guiding Educational Reform; SANICS, Self-Assessment Nursing Informatics Competency Scale.

**Table 3:** Summary of measurement properties of instruments included

Empty cells indicate no available results for measurement properties																														
Instrument	Content validity				Instrument development	Structural validity				Internal consistency				Reliability				Criterion validity				Hypotheses testing				Responsiveness				Recommended grade
	Number of studies	ROB score	Rating	Quality of evidence (GRADE)	Quality of evidence (GRADE)	Number of studies	ROB score	Rating	Quality of evidence (GRADE)	Number of studies	ROB score	Rating	Quality of evidence (GRADE)	Number of studies	ROB score	Rating	Quality of evidence (GRADE)	Number of studies	ROB score	Rating	Quality of evidence (GRADE)	Number of studies	ROB score	Rating	Quality of evidence (GRADE)	Number of studies	ROB score	Rating	Quality of evidence (GRADE)	
Nursing informatics (NI) competence instruments for nurses																														
Canadian Nurse Informatics Competency Assessment Scale (C-NICAS), English version ^51^						1	A	+	M	1	V	+	H																	B
Canadian Nurse Informatics Competency Assessment Scale (C-NICAS), French version ^46^	1	D	+	M						1	D	?	VL																	B
Korean Nursing Informatics Competence Assessment Scale (K-NICAS) ^44^	1	D	+	M	D	1	A	−	M	1	V	?	H					1	D	−	L	1	V	+	H					B
Nursing Informatics Competencies Questionnaire (NICQ) ^42^	1	D	+	M	D	1	I	−	VL	1	V	?	H																	B
Nursing Informatics Competency Assessment Tool (NICAT) ^45^	1	D	+	M	D																									B
Self-Assessment of Nursing Informatics Competency Scale-18 (T-SANICS 18-item) ^49^	1	D	+	M		1	V	−	H	1	V	?	H																	C
The Arabic Self-assessment Nursing Informatics Competency Scale (A-SANICS 30-item) ^48^	1	D	+	M		1	A	+	M	1	V	+	H									1	V	+	H					A
TIGER-based Assessment of Nursing Informatics Competencies (TANIC) ^16^ , ^52^	1	D	+	M	D					1	D	?	L																	B
Turkish version of the TIGER-based Assessment of Nursing Informatics Competencies (T-TANIC) ^47^	1	D	+	M		1	A	+	M	1	V	+	H	1	A	+	VL													A
Unnamed ^50^	1	D	?	M						1	D	?	L																	B
Unnamed ^43^	1	D	+	M	D	1	I	+	VL	1	V	?	H	1	D	?	VL					1	A	+	M					B
Nursing informatics (NI) competence instruments for nursing students																														
Canadian Nurse Informatics Competency Assessment Scale-Version 2 (C-NICAS-V2) ^55^	1	D	?	M		1	A	+	M	1	V	+	H																	B
Knowledge, Skills, and Attitudes toward Nursing Informatics (KSANI) Scale ^53^					D	1	A	+	M	1	V	+	H																	B
Korean Self-Assessment of Nursing Informatics Competencies Scale (K-SANICS 30-item) ^54^	1	D	+	M		1	A	?	M	1	V	?	H									1	D	+	L					B
SANICS 30-item ^38^ , ^39^ , ^40^						3	A–I–A	±		3	V	?	M									1	D	+	L	3	D–I–I	+	L	B
Self-Assessment of Nursing Informatics Competencies Scale (SANICS 18-item) ^56^						1	A	?	M	1	V	?	H																	B
NI instruments for nurse leaders																														
Nursing Informatics Competency Assessment for the Nurse Leader (NICA-NL) ^57^ , ^58^	1	D	+	M	D	1	A	?	M	1	V	?	H																	B
NI competence instruments for informatics nurse specialists																														
The Nursing Informatics Competency Assessment L3/L4 (NICA: L3/L4) ^59^	1	D	+	M						1	V	?	M																	B
Digital health competence instruments																														
DigiComInf ^60^	1	D	+	M	D	1	A	+	M	1	V	+	H																	A
DigiHealthCom ^60^	1	D	+	M	D	1	A	+	M	1	V	+	H																	A
Digital Competence Questionnaire (DCQ) ^61^ , ^63^	1	D	+	M	D	1	A	+	M	1	V	+	H																	A
Knowledge, attitudes, and practices (KAP) on digital health) ^62^	1	D	+	M	I					1	I	?	VL	1	A	+	VL													B
The digital competence test ^41^					D					1	D	?	L									1	D	+	L					B
Unnamed ^64^						1	V	+	H	1	V	+	H									1	V	+	H					B
Attitudes toward information and communication technology instruments																														
Shortened version-Information Technology Attitude Scales for Health (ITASH-sv) ^65^	1	D	+	M		1	V	+	H	1	V	+	H									1	V	+	H					A
Technology Attitude Survey ^66^						1	A	+	M	1	V	+	H																	B
Turkish Version of the Technology Attitude Survey ^67^						1	A	+	M	1	I	+	VL																	B

Notes:

• ROB score (COSMIN: consensus-based standards for the selection of health measurement instruments): V, very good; A, adequate; D, doubtful; I, inadequate.

• Quality (modified GRADE: grading of recommendations assessment, development and evaluation): H, high; M, moderate; L, low; VL, very low.

• Criteria for content validity rating: overall content validity is sufficient (+), insufficient (−), inconsistent (±), indeterminant (?).

• Criteria for “other measurement properties” rating: measurement property rating: (+), sufficient; (−), insufficient; (?), indeterminate.

• Recommended grade: (A) instruments with evidence of sufficient content validity (at any level) and at least low-quality evidence of sufficient internal consistency (recommended for use); (B) instruments not falling under categories (A) or (C; potentially recommendable); (C) instruments with high-quality evidence of an insufficient measurement property (not recommended for use).

#### Nursing Informatics Instruments for Nurses

Instrument development studies were reported for only five instruments, 16 42 43 44 45 and all were rated as being of “doubtful” quality. Nine 16 42 43 44 45 46 47 48 49 out of 11 instruments had studies reporting on their content validity, which showed “moderate” quality of evidence, except for one instrument, 50 which was rated as indeterminate because no results were reported on item relevance, comprehensiveness, or comprehensibility.

Seven instruments reported on their structural validity. Four of these 43 47 48 51 were rated as sufficient, with a “moderate” quality of evidence, except for one, 43 which had a very low quality of evidence due to an inadequate sample size for confirmatory factor analysis. The remaining three instruments 42 44 49 were rated as insufficient because of inadequate sample sizes or unmet confirmatory factor analysis criteria, although the quality of evidence varied across studies.

Almost all instruments, except one, evaluated their internal consistency. For most of them, 42 43 44 46 49 50 52 this measurement property was rated as indeterminate, primarily due to insufficient or missing structural validity data, while for three instruments 47 48 51 it was rated as sufficient with a “high” quality of evidence.

Construct validity was reported for three instruments, 43 44 48 all receiving a sufficient rating with a “high” or “moderate” level of evidence. Reliability was assessed in two instruments, inter-rater and test–retest, respectively, and, for both, the level of evidence was “very low” due to a single study with a small sample size 43 47 and the statistical test used. 43 Finally, criterion validity was reported as insufficient for one instrument. 44

Overall, the measurement properties of most instruments revealed limited evidence. Most instruments showed moderate evidence for content validity, while results for structural validity and internal consistency were mixed, often affected by inadequate sample sizes. Construct validity was supported for a few instruments with moderate to strong evidence, whereas reliability and criterion validity showed very low or insufficient evidence. Notably, two instruments 47 48 demonstrated sufficient measurement properties across key domains.

#### Nursing Informatics Instruments for Nursing Students

A development study was reported for only one instrument 53 and was evaluated as being of “doubtful” quality of evidence. Two instruments reported on their content validity, which was rated as sufficient for one 54 and indeterminate 55 for the other due to missing information on item relevance, comprehensiveness, and comprehensibility.

Structural validity was evaluated for all instruments. This property was rated as sufficient in two instruments 53 55 and indeterminate in two others 54 56 due to missing model fit indices 56 and the presence of only two items per dimension, 54 with a “moderate” level of evidence. For one instrument 38 39 40 structural validity was assessed in three different samples and rated as inconsistent because of differing item dimensions across studies and the absence of confirmatory factor analysis.

Internal consistency was reported for all instruments. Two instruments 53 55 demonstrated sufficient internal consistency with a “high” quality of evidence, while three other instruments 38 39 40 54 56 one of which was tested in three different samples 38 39 40 showed indeterminate results.

Construct validity was reported for two instruments 39 54 and was rated as sufficient, although with a “low” level of evidence due to the doubtful methodological quality of the studies, mainly related to suboptimal statistical methods. Finally, responsiveness was evaluated for one instrument and rated as sufficient in three different samples 38 39 40 but the level of evidence was “low” because of doubtful or inadequate methodological quality across these studies.

Overall, the instruments in this group showed limited and partly inconsistent evidence regarding their measurement properties. Development and content validity were rarely and inconsistently reported, while structural validity and internal consistency showed mixed results. Construct validity and responsiveness were evaluated in a few studies and supported by low to moderate quality of evidence.

#### Nursing Informatics Instruments for Nurse Leaders

This group comprised one instrument. Its measurement properties were assessed in two studies. 57 58 The development study was rated as of “doubtful” methodological quality, and the evidence for its content validity was of “moderate” quality. 57 Structural validity was indeterminate due to incomplete information required for a sufficient rating, and two dimensions included only two items each. 58 Consequently, internal consistency was also indeterminate, as criteria for at least low evidence for sufficient structural validity were unmet. Overall, evidence for this instrument's measurement properties was limited, with indeterminate structural validity and internal consistency.

#### Nursing Informatics Instruments for Informatics Nurse Specialists

Only one instrument was included in this group. Evidence for content validity was of “moderate” quality, while internal consistency was indeterminate due to missing structural validity data. 59 Overall, evidence for this instrument's measurement properties was limited.

### Digital Health Competence Instruments

Six instruments were identified as measuring digital health competencies. Two instruments assessed these competencies only in nurses, while the other four instruments assessed them in healthcare professionals, including nurses. Most of the instruments were tested in samples from Europe ( *n* = 3), and one instrument was tested in an international sample of nurses. See Tables 2 and 3 for a detailed summary of the instruments' characteristics and their measurement properties (additional information on measurement values is available in Supplementary File , available in the online version only).

Five out of six instruments reported a development study that was rated as of “doubtful” quality for most of them 41 60 61 and “inadequate” for one, 62 due to an unclear construct description. Four 60 61 62 of these five instruments also reported a content validity study supported by “moderate” evidence. Four instruments evaluated their structural validity, with “moderate” 60 63 and “high” 64 quality of evidence. Internal consistency was assessed for all instruments; for four 60 63 64 of them the quality of evidence was “high,” while for two 41 62 it was indeterminate due to unknown structural validity.

Construct validity was also demonstrated as sufficient for two instruments, although the level of evidence was “high” for one 64 and “low” 41 for the other, due to a “doubtful” study (poor description of subgroup characteristics). Finally, test-retest reliability was assessed in one instrument 62 with very “low” evidence, due to an inadequate sample size.

Overall, the instruments showed mainly moderate to high evidence for content, structural validity, and internal consistency, while development studies, construct validity, and test–retest reliability were supported by weaker or insufficient evidence. Notably, three instruments 60 61 63 demonstrated sufficient measurement properties across content validity, structural validity, and internal consistency.

### Attitudes Toward Information and Communication Technology Instruments

Three instruments were identified as measuring attitudes toward information and communication technology. All instruments assessed these competencies in nursing students across three different countries. Tables 2 and 3 provide a detailed overview of the instruments' characteristics and measurement properties (for further details on measurement values, please refer to Supplementary File , available in the online version only).

A content validity study was reported for one instrument 65 with “moderate” quality of evidence, while for another instrument 66 it was mentioned, but since the study did not ask professionals about the relevance, comprehensiveness, or comprehensibility of the survey items, it was not regarded as a content validity study according to the COSMIN checklist. Structural validity and internal consistency of all three instruments were rated as sufficient, with “moderate” 66 67 or “high” 65 quality of evidence for structural validity, and “high” 65 66 or “very low” 67 evidence for internal consistency. For one instrument, 65 construct validity was also demonstrated as sufficient with “high” quality of evidence.

Overall, the instruments in this group showed sufficient measurement properties, with moderate to strong evidence for structural validity and internal consistency. However, evidence for content validity was available for only one instrument. 65

## Discussion

Numerous studies on NI, digital health, and ICT competencies have been conducted in diverse contexts, highlighting varying competence requirements depending on factors such as nursing role, position, and experience (e.g., nurse, nursing student, nurse leader). These studies have also contributed to the development of various competence measurement instruments. Additionally, several instruments have been created in different countries, reflecting the variability of competencies across healthcare and educational settings.

Regarding their measurement properties, we categorized the instruments according to the COSMIN methodology 35 to formulate recommendations for their use. The 27 identified instruments were categorized into three groups: (A) instruments with evidence of sufficient content validity (at any level) and at least low-quality evidence of sufficient internal consistency; (B) instruments not falling under categories (A) or (C); (C) instruments with high-quality evidence of an insufficient measurement property. Six instruments fell into category (A) the DigiHealthCom and DigiComInf instruments, 60 the Turkish version of the TIGER-based Assessment of Nursing Informatics Competencies (T-TANIC), 47 the shortened version of the Information Technology Attitude Scales for Health (ITASH-sv), 65 the Digital Competence Questionnaire (DCQ), 61 63 and the Arabic Self-Assessment Nursing Informatics Competency Scale (A-SANICS 30-item version). 48 The Turkish Self-Assessment of Nursing Informatics Competency Scale-18 (T-SANICS 18-item version) 49 was placed in category (C). The remaining 20 instruments were categorized under (B).

### Category A Instruments

The six instruments in category A can be recommended for use, and the results obtained from these instruments can be considered reliable. Three of these instruments, the DigiHealthCom, DigiComInf, 60 and DCQ, 61 63 measure digital health competencies. The T-TANIC 47 and A-SANICS 48 assess NI competencies, while the ITASH-sv 65 evaluates attitudes toward ICT. The DCQ is a short questionnaire (12 items) with good feasibility, as it is brief. Furthermore, it was tested in an international sample of nurses, enhancing its generalizability. However, additional measurement evaluations are needed to strengthen its validity and reliability, including confirmatory factor analysis, test-retest reliability, construct validity, and responsiveness. The DigiHealthCom and DigiComInf instruments have the advantage of being applicable to various healthcare professionals and exploring factors influencing digital health competence. Nonetheless, both instruments have been validated only in Finland, and, as with the DCQ, further measurement testing is required. The T-TANIC and A-SANICS are translated and adapted versions of the original instruments, tested in samples of nurses in Turkey and Jordan, respectively. The T-TANIC was also assessed for inter-rater reliability; however, further studies with larger sample sizes are needed to strengthen the level of evidence for this property. One possible limitation of the T-TANIC is its feasibility, as it is a lengthy instrument consisting of 85 items. The A-SANICS demonstrated “high” quality of evidence for its construct validity, including convergent and discriminant validity. The ITASH-sv was tested in a sample of nursing students in South Korea and also showed “high” quality of evidence for construct validity, both convergent and discriminant. Overall, although all the instruments are recommended for use, they should be tested in longitudinal studies with probability samples to assess their responsiveness, for example, in the context of educational programs aimed at improving these competencies.

### Category B Instruments

Instruments categorized as B have the potential to be recommended for use, but further research is needed to evaluate their measurement quality. For example, the SANICS 30-item 38 39 40 is the only instrument with multiple studies assessing its measurement properties; however, none of these studies reported content validity, and the other measurement properties were either insufficient or of “low” quality of evidence. Interestingly, although the original SANICS 30-item did not demonstrate adequate measurement properties, the A-SANICS 48 was classified as a category A instrument. This discrepancy may be explained by the fact that the latter was tested in a sample of nurses rather than nursing students. Some other instruments in this category, despite showing adequate measurement properties in some aspects, either lacked information on content or structural validity or reported structural validity as insufficient or indeterminate.

To improve the instruments under this category, future research should assess content validity, confirm structural validity through confirmatory factor analysis, and evaluate reliability (e.g., internal consistency, test–retest). Responsiveness should be tested in longitudinal studies. Validation in diverse and larger samples is also recommended.

### Category C Instruments

The T-SANICS 18-item 49 was placed under this category and is not recommended for use. Its structural validity received a “high” quality of evidence for an insufficient rating: some fit indices were below the acceptable threshold; moreover, one dimension includes only two items, one of which has a factor loading below 0.70, and several items in other dimensions show factor loadings well below 0.30. To improve this instrument, revisions should address its weak structural validity by refining low-loading items, expanding underdeveloped dimensions, and then reassessing its factor structure through robust measurement testing.

### Overall Discussion

To better contextualize our findings, we compared them with those of a recent systematic review by Li et al. 31 The literature review conducted by Li et al 31 focused exclusively on instruments related to NI, while our review adopted a broader perspective, encompassing instruments addressing the wider concepts of ICT and digitalization in nursing. Therefore, comparisons can only be made between these specific instruments. All NI-related instruments included in their review were also assessed in ours, whereas some other instruments in Chinese were excluded from our review due to our inclusion criteria.

Although both reviews classified most NI instruments in category B, notable discrepancies were found in the evaluation of four tools. Specifically, Li et al 31 rated the Nursing Informatics Competencies Questionnaire (NICQ), 42 the Korean Nursing Informatics Competence Assessment Scale (K-NICAS), 44 and the Korean Self-Assessment of Nursing Informatics Competencies Scale (K-SANICS) 54 as category C (not recommended for use), whereas we classified them as category B. These differences stem from divergent applications of the COSMIN methodology.

In the case of the NICQ, both reviews agreed on a rating of “inadequate” for structural validity. According to COSMIN guidelines, this should result in a “very low” overall quality of evidence. Nevertheless, Li et al 31 rated it as “high,” a conclusion not methodologically justifiable, as even a single study with serious risk of bias requires downgrading by up to three levels. 36

Regarding the K-NICAS, we rated the methodological quality as “adequate,” whereas Li et al 31 rated it “very good.” This disagreement arose from differing interpretations of sample size: our review judged the sample inadequate, as it did not meet COSMIN's recommendation of seven respondents per item. 36

Similarly, in the case of the K-SANICS, both reviews rated the structural validity as “adequate.” Nevertheless, Li et al 31 did not downgrade the quality of evidence from “high” to “moderate,” despite the fact that “there was only one study of adequate quality,” 36 which would warrant such a downgrade.

Finally, the T-TANIC 47 was rated as category B in the Chinese review and as category A in ours. This difference stems from diverging evaluations of content validity. Although both reviews rated the methodological quality as “doubtful,” we considered the results “sufficient,” while Li et al 31 rated them as “inconsistent.” COSMIN acknowledges the subjectivity involved in evaluating content validity, especially when the evidence is incomplete or mixed, but also emphasizes its central role in instrument quality. 37

Overall, our review identified common weaknesses in the development and content validation processes of many instruments. These include limited use of qualitative methods, over-reliance on translations, and frequent use of only quantitative indicators like the content validity index. Such limitations underscore the need for closer adherence to the COSMIN checklist and the involvement of measurement experts. Finally, we note that only one objective instrument (performance-based test) was identified, reinforcing the need to develop tools that go beyond self-reporting and allow for a more comprehensive and unbiased assessment of digital competencies. This limitation was also highlighted by Mainz et al. 4 Therefore, there is a need for updated, theory-based, and performance-oriented instruments that move beyond technical skills, knowledge, and self-assessment approaches. 4 Such instruments would better capture the multidimensional nature of digital competence in nursing practice. Moreover, the publication period of the included instruments should be considered, as some were developed several years ago and may not fully reflect the digital skills required in contemporary healthcare practice.

### Limitations

This review is limited to English-language publications, possibly excluding relevant studies in other languages. However, its strengths include multiple search strategies, such as citation tracking and expert consultation, enhancing comprehensiveness. Another possible limitation of this review is that three of the included instruments were unnamed; although this does not affect their measurement properties, the absence of specific names may limit other researchers' ability to fully identify and evaluate them. In addition, several instruments were developed outside of Canada and the United States, which may affect their cross-cultural applicability and limit the generalizability of the findings to North American contexts.

## Conclusion

This review highlights that, despite growing attention to digital transformation in healthcare, evidence on the psychometric quality of instruments assessing nurses' digital competencies remains limited. Improving nurses' digital competencies is crucial for effective collaboration and care coordination, especially in resource-limited settings. 64 Increasing focus on these competencies supports public health and nursing education goals. 68 Valid, reliable instruments are essential for assessing and monitoring these competencies through education and training. 19 Nurse educators are key in integrating digital skills into curricula aligned with core competencies.

The findings emphasize the use of valid instruments to guide education and workforce development in nursing digital competencies. Measuring nurses' digital competencies with robust instruments, including not only self-report measures but also performance-based tests, helps identify training needs, guide support, and promote the integration of informatics into clinical practice.

## Notes

**Clinical Relevance Statement**
As healthcare increasingly embraces digital transformation, it is essential to ensure that nurses possess the necessary digital competencies to deliver safe and effective care. This review addresses the critical need for valid and reliable tools to assess these competencies, aiding in the enhancement of clinical practice. By identifying and evaluating available assessment instruments, the findings guide the selection of context-appropriate tools and emphasize the importance of integrating digital competence evaluations into both nursing education and everyday clinical workflows.
**Multiple-Choice Questions**

1. What method was used to assess the measurement properties of instruments assessing digital competence in this review?
  - Delphi method
  - PRISMA guidelines
  - COSMIN criteria
  - SWOT analysis
  **Correct Answer:** The correct answer is option c. The COSMIN methodology is specifically designed to evaluate the methodological quality of studies on measurement properties, such as validity, reliability, and responsiveness. In systematic reviews focusing on the evaluation of instruments, COSMIN provides a structured and rigorous framework for assessing psychometric quality.
2. Of all the instruments included in this review, which were the ones recommended for use?
  - All instruments assessing nursing informatics and ICT.
  - The six instruments categorized under group A.
  - Only the instrument categorized under group C.
  - All instruments categorized under group B.
  **Correct Answer:** The correct answer is option b. Because, following the COSMIN methodology, instruments were grouped based on the quality of their measurement properties. Only the instruments placed in category A met sufficient criteria to be recommended for use without reservation.

## Notes

**Corrigendum:** This article was updated as per the correction note published on March 19, 2026. Doi of the corrigendum is 10.1055/a-2815-8240.
