# Supplementary material for: Measurement Properties of Instruments Assessing Digital Competence in Nursing: A Systematic Review
Source: Appl Clin Inform. 2026 Jan 22;17(1):1–18. doi: 10.1055/a-2780-7093 (PMC12826851; doi:10.1055/a-2780-7093)
Supplement: Supplementary file 1 — Supplementary Material [file 10-1055-a-2780-7093_27935128.pdf]

Thieme

Supplementary File: Reliability, Validity, and Responsiveness Values

Empty cells indicate no available results for measurement properties.

Instrument	Structural validity	Internal consistency	Reliability	Criterion validity	Hypotheses testing	Responsiveness
	Summary or pooled result	Summary or pooled result	Summary or pooled result	Summary or pooled result	Summary or pooled result	Summary or pooled result
Nursing Informatics (NI) Competence Instruments for Nurses						
**Canadian Nurse Informatics Competency Assessment Scale (C-NICAS), English version[51]**	EFA<br>4 dimensions<br><br>Eigenvalues > 1<br>Total variance explained = 60.99%	Cronbach's α = 0.81-0.92				
**Canadian Nurse Informatics Competency Assessment Scale (C-NICAS), French version[46]**		Cronbach's α = 0.74				
**Korean Nursing Informatics Competence Assessment Scale (K-NICAS)[44]**	CFA<br>5 dimensions<br><br>CFI = 0.93, RMSEA = 0.06; TLI = 0.92	Cronbach's α = 0.73-0.85		r < .70	The result is in accordance with the hypothesis<br><br>AVE = 0.55 – 0.83	
**Nursing Informatics Competencies Questionnaire (NICQ)[42]**	EFA<br>3 dimensions<br><br>Factors loadings < 0.40<br>Total variance explained = 44.9%	Cronbach's α = 0.94-0.98				
**Self-Assessment of Nursing Informatics Competency Scale-18 (T-SANICS 18-item)[49]**	EFA<br>CFA<br>3 dimensions<br><br><br>Factors loadings < 0.30<br><br>CFI = 0.92, RMSEA = 0.07; TLI = 0.97, GFI = 0.92	Cronbach's α = 0.67-0.92				
**The Arabic Self-assessment Nursing Informatics Competency Scale (A-SANICS 30-item)[48]**	EFA<br>CFA<br>5 dimensions<br><br>Total variance explained = 71.62%<br><br>CFI = 0.916, RMSEA = 0.075; TLI = 0.901, SRMR= 0.070, IFI = 0.917	Cronbach's α = 0.959 - 0.984			The result is in accordance with the hypothesis<br><br>AVE = 0.521 – 0.665	
**TIGER-based Assessment of Nursing Informatics Competencies (TANIC)[16,52]**		Cronbach's α = 0.91-0.99				
**Turkish version of the TIGER-based Assessment of Nursing Informatics Competencies (T-TANIC)[47]**	EFA<br>3 dimensions<br><br>Factors loadings > 0.40<br>Total variance explained = 65.39%	Cronbach's α = 0.94-0.98	ICC = 0.94 – 0.98			
**Unnamed Scale[50]**		Cronbach's α = 0.98				
**Unnamed scale[43]**	CFA<br>3 dimensions<br><br>CFI = 0.99, RMSEA = 0.051	Composite reliability < 0.70	Spearman's rank correlation-coefficient (ρ) > 0.90		The result is in accordance with the hypothesis<br><br>AVE = 0.61 – 0.95	
Nursing Informatics (NI) Competence Instruments for Nursing students						

**Canadian Nurse Informatics Competency Assessment Scale-Version 2 (C-NICAS-V2)[55]**	EFA<br>4 dimensions<br><br>Total variance explained = 55.10%	Cronbach's α = 0.81-0.82				
**Knowledge, Skills, and Attitudes towards Nursing Informatics (KSANI) Scale[53]**	EFA<br>4 dimensions<br><br>Factors loadings > 0.40<br>Eigenvalues > 1<br>Total variance explained = 57.9%	Cronbach's α = 0.79-0.86				
**Korean Self-Assessment of Nursing Informatics Competencies Scale (K-SANICS 30-item)[54]**	EFA<br>6 dimensions<br><br>Factors loadings > 0.40<br>Total variance explained = 70.90%	Cronbach's α = 0.64-0.93			The result is in accordance with the hypothesis	
**SANICS 30-item[38,39,40]**	EFA<br>5 dimensions<br><br>Summarized Factors loadings = 0.40 - 0.68<br>Total variance explained > 65.0%	Cronbach's α = 0.84-0.95			The result is in accordance with the hypothesis	SRM = 0.99
**Self-Assessment of Nursing Informatics Competences Scale (SANICS 18-item)[56]**	Mokken scaling analysis<br>1 dimension	Cronbach's α = 0.93				
NI Instruments for Nurse Leaders						
**Nursing Informatics Competency Assessment for the Nurse Leader (NICA-NL)[57,58]**	EFA<br>6 dimensions<br><br>Factors loadings > 0.398	Cronbach's α = 0.81-0.96				
NI Competence Instruments for Informatics Nurse Specialists						
**The Nursing Informatics Competency Assessment L3/L4 (NICA - L3/L4)[59]**		Cronbach's α = 0.91-0.99				
Digital Health Competence Instruments						
**DigiComInf[60]**	EFA<br>3 dimensions<br><br>Eigenvalues > 1<br>Total variance explained = 59.60%	Cronbach's α = 0.74-0.88				
**DigiHealthCom[60]**	EFA<br>5 dimensions<br><br>Eigenvalues > 1<br>Total variance explained = 68.86%	Cronbach's α = 0.91-0.97				
**Digital Competence Questionnaire (DCQ)[61,63]**	EFA<br>2 dimensions<br><br>Total variance explained = 57.00%	Cronbach's α = 0.81-0.84				
**Knowledge, attitudes, and practices (KAP) on digital health[62]**		Cronbach's α = 0.76	ICC = 0.76 – 0.99			
**The Digital competence test[41]**		Greatest lower bound = 0.66			The result is in accordance with the hypothesis	

**Unnamed scale**[64]	EFA<br>CFA<br>2 dimensions<br><br>CFI = 0.960	Cronbach's α = 0.82-0.88			The result is in accordance with the hypothesis<br><br>AVE = 0.543	
Attitudes towards Information and Communication Technology Instruments						
**Shortened version-Information Technology Attitude Scales for Health (ITASH)**[65]	CFA<br>4 dimensions<br><br>CFI > 0.95, RMSEA < 0.06	Ordinal coefficient α = 0.75- 0.90			The result is in accordance with the hypothesis<br><br>AVE = 0.52 – 0.62	
**Technology Attitude Survey**[66]	EFA<br>2 dimensions<br><br>Eigenvalues > 1<br>Total variance explained = 63.50%	Cronbach's α = 0.92-0.95				
**Turkish Version of the Technology Attitude Survey**[67]	EFA<br>2 dimensions<br><br>Factors loadings > 0.70	Cronbach's α = 0.90-0.91				

AVE: Average Variance Extracted; **CFA**: Confirmatory Factor Analysis; **CFI**: Comparative Fit Index; **EFA**: Exploratory Factor Analysis; **GFI**: Goodness of Fit Index; **ICC**: Intraclass Correlation Coefficient; **IFI**: Incremental Fit Index; **RMSEA**: Root Mean Square Error of Approximation; **SRM**: Standardized Response Mean; **SRMR**: Standardized Root Mean Square Residual; **TLI**: Tucker-Lewis Index;
